# Detection of femtosecond spin injection into a thin gold layer by time and spin resolved photoemission

**DOI:** 10.1038/s41598-020-69477-y

**Published:** 2020-07-28

**Authors:** K. Bühlmann, G. Saerens, A. Vaterlaus, Y. Acremann

**Affiliations:** Laboratory for Solid State Physics, ETH Zurich, 8093 Zurich, Switzerland

**Keywords:** Spintronics, Ferromagnetism

## Abstract

The ultrafast demagnetization effect allows for the generation of femtosecond spin current pulses, which is expected to extend the fields of spin transport and spintronics to the femtosecond time domain. Thus far, directly observing the spin polarization induced by spin injection on the femtosecond time scale has not been possible. Herein, we present time- and spin-resolved photoemission results of spin injection from a laser-excited ferromagnet into a thin gold layer. The injected spin polarization is aligned along the magnetization direction of the underlying ferromagnet. Its decay time depends on the thickness of the gold layer, indicating that transport as well as storage of spins are relevant. This capacitive aspect of spin transport may limit the speed of future spintronic devices.

## Introduction

A network of linear components in electronics (consisting of resistors, capacitors, inductors and transformers) can be fully described as a linear multi-port structure. If we investigate its behavior by studying the direct current (DC) transport characteristics the circuit will look as if it only consisted of resistors: At a frequency of 0 Hz, there is no current flowing through the terminals of the capacitors and the voltage drop across coils is zero. Only if we study the *dynamics* of the circuit, the inductors, capacitors and transformers start to become visible. In this paper we discuss the capacitive aspect of spin transport in a thin gold layer and experimentally detect the injection and storage of spins with femtosecond time resolution.

Static spin transport experiments can be described by spin-dependent resistances. The current can be separated into two components of opposite spin. These current components are affected by a spin-dependent resistance along the current path. Spin flips are modeled by conductance between the two spin channels^1^. This “two-current model” led to the explanation of the giant magnetoresistance (GMR) effect^2, 3^ and the tunnel magnetoresistance (TMR) effect^4, 5^, which are routinely applied in modern hard disk read heads. Besides sensor applications, spin currents can be used to manipulate the magnetization of a ferromagnetic data storage element^6–10^.

In static transport we need the concept of spin accumulation in order to describe the splitting of the spin-dependent chemical potentials^1^. However, the actual *storage* of spin angular momentum is not visible in the DC transport properties. The number of accumulated spins per volume and the spin splitting of the chemical potentials $\Delta \mu = \mu _\uparrow - \mu _\downarrow$ can be seen as a spin accumulation capacitance density. This quantity has been introduced by Zhu et al.^11^. The spin capacitance tells us, how much spin accumulation per volume is achieved per spin voltage unit and is the equivalent to the capacitance density in electronics. In this work, we use spin and time resolved photoemission to directly detect the spin accumulation of injected spins in a gold layer.

The spin currents are generated by the ultrafast demagnetization of a ferromagnet^12^: a ferromagnet is exposed to a femtosecond laser pulse; if the ferromagnet is in contact with a nonmagnetic metal, a spin current is injected into the non-magnet^13–15^. Another way of generating femtosecond spin current pulses is by optical pumping of a heavy metal with circularly polarized light^16^.

Recent time-resolved experiments^17–19^ have demonstrated the detection of spin injection using the linear, complex and second harmonic magneto-optic Kerr effect (SHMOKE). The authors of^18, 19^ observed the generation of non-thermal spin transport, as predicted theoretically^20–22^. SHMOKE is sensitive to spin polarization at interfaces and is therefore well suited for observing transport effects. Multiple interfaces can contribute to the measured signal, and so the spin current generated by a particular interface cannot easily be determined. The spin current can be detected by observing the THz radiation emitted through the spin-Hall effect^23, 24^.

The most direct way of measuring the spin polarization in a metal is by spin-resolved photoelectron spectroscopy. This method does not rely on the spin-orbit coupling within the solid and is not affected by pump-induced changes of the optical properties of the solid. If the sample is probed by UV radiation, photoemission is surface sensitive and therefore provides exclusive access to the topmost layer of the sample. It is therefore not affected by the spin dynamics at other interfaces. However, detecting a small spin-current-induced polarization by time-resolved photoemission is challenging. Only recently have highly efficient spin detectors become available, which offer parallel detection of the spin and energy of electrons^25–28^.

**Figure 1:**
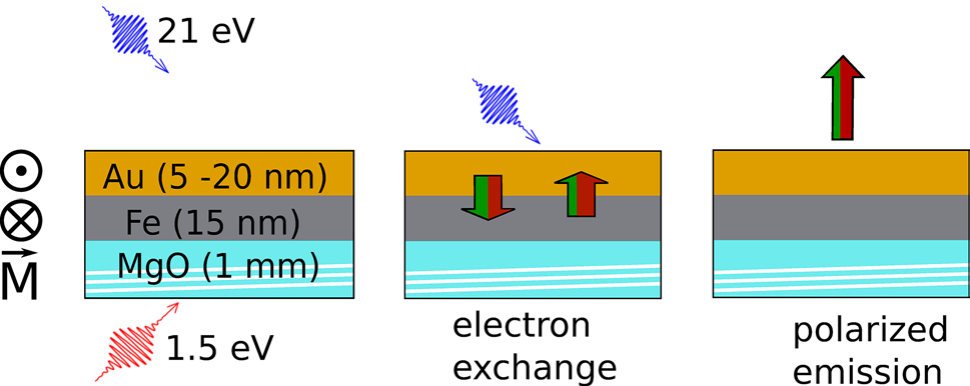
Schematic representation of the pump-probe geometry for transport experiments. The sample is deposited onto a transparent MgO substrate without breaking vacuum during transport to the photoemission chamber. The first layer consists of the magnetic Fe layer. It is covered by a Au layer of variable thickness. An alternating magnetic field pulse of 100 Oe is applied to the sample, magnetizing it along the magnetization direction *M*. The measurement itself is performed in remanence. The sample is excited by the s-polarized pump laser from the back side through the MgO substrate (red arrow). The ultraviolet probe pulse (p-polarized, shown as a blue arrow) detects the induced spin polarization in the Au layer by spin-resolved photoemission. The red-green arrows indicate, that the same number of electrons are transported from the Au layer to the Fe layer as from the Fe layer to the Au layer as charge neutrality is maintained. However, the majority electrons (indicated in red) transported from the Fe to the Au layer outnumber the minority electrons (indicated in green).

## Methods

To probe the spin dynamics with a femtosecond time resolution, we perform a laser-pump–ultraviolet-probe experiment in a back-pump geometry. The method is illustrated in Fig. 1. A 15 nm thick iron film is grown on a MgO (001) substrate at room temperature. It is followed by a gold layer of variable thickness. The magnetic easy axis is along the [110] direction of Fe. The pressure in the deposition- and measurement vacuum chamber is $< 10^{-10}\,\mathrm {mbar}$. The sample is transported between these chambers without breaking vacuum.

The measurement setup consists of an amplified tabletop Ti:sapphire laser system (Coherent Inc. Legend Elite), delivering pulses of 20 fs FWHM duration at a 10 kHz repetition rate, a pulse energy of 1 mJ and 800 nm wavelength. The sample is excited by the pump pulses through the transparent substrate at a pump fluence of $4\,\text {mJ/cm}^2$ and a beam diameter of 1 mm. Upon excitation, the sample surface is probed by the radiation of a higher harmonic generation source operating at 21 eV^29–31^. The probe beam has a diameter of $\approx 0.5\,\mathrm {mm}$. In order to avoid systematic errors arising from slow drifts (for example of the spatial overlap between the pump- and probe beams), we perform several short time scans, which are added up during data analysis. In addition, active beam position stabilization is used on the pump- and probe beams before frequency conversion.

The photoelectrons are mainly emitted from the gold surface: According to^32^ the inelastic mean free path for electrons at a kinetic energy of 21 eV is $<1\,\mathrm {nm}$. The emitted electrons are energy filtered by a hemispherical energy analyzer (Specs Phoibos 150). As we detect electrons at close to the Fermi edge, inelastically scattered electrons are suppressed. At the output of the hemishpere the electron spin is analyzed by a spin-polarized low-energy electron diffraction (SPLEED) setup^25–27^ by reflecting the electrons off an Ir (001) crystal covered with one monolayer of Au. For detection, the reflected electrons are incident upon a micro channel plate (MCP), which is then imaged by a CCD camera. The image on the detector contains the energy information along the dispersive direction. The spin information is contained in the reflectivity of the analyzer crystal. Therefore, we reverse the magnetization of the ferromagnetic layer $M_{\uparrow , \downarrow }$ by a pulsed coil. During the measurement, the sample is in its remanent state. From two sets of images at $M_{\uparrow , \downarrow}$ the polarization can be obtained by calculating the asymmetry, and correction by the Sherman function^33^. Further details of the electron spectrometer and spin filter are described in^34^.

**Figure 2:**
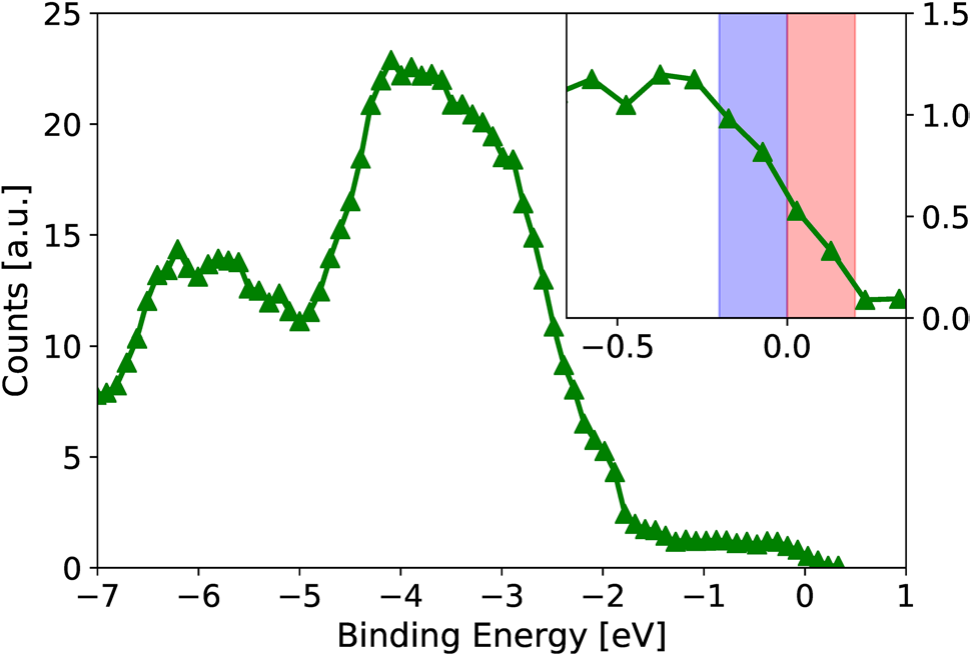
Spin-integrated photoemission spectrum from the Au layer: the 5d levels (at a binding energy of −4 eV and −6 eV) provide a significantly stronger photoemission yield compared to the valence band. The inset shows the sections around the Fermi energy (normalized to 0 eV), referred to as “below $E_f$” (blue) and “above $E_f$” (red) throughout the manuscript.

Figure 2 shows the spin-integrated photoemission spectrum from the sample. To observe transport effects, we focus on the Fermi edge. The states of interest at the Fermi energy $E_f$ are significantly less intense than the d-band peaks. The inset shows the spectrum around $E_f$ (normalized to 0 eV in the interval $\pm 0.2$ eV around $E_f$), labeled as “below $E_f$” (blue) and “above $E_f$” (red) throughout the manuscript. The energy interval is given by the energy bandwidth of the spin detector.

**Figure 3:**
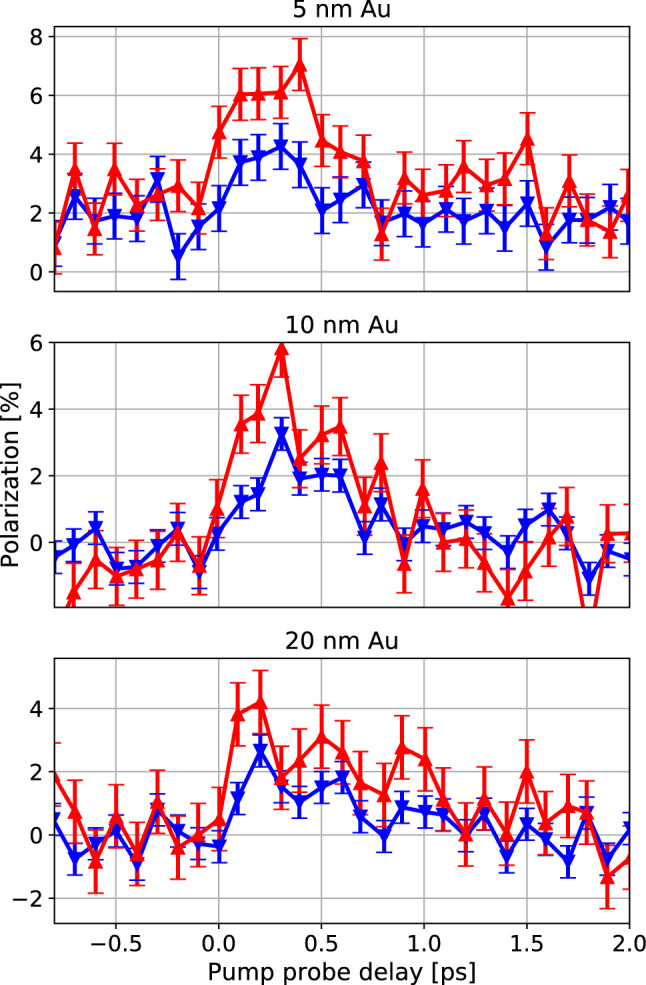
Measured spin polarization on the Au film surface as a function of the pump-probe delay. For states both below (blue) and above $E_f$ (red), we observe a spin-current-induced spin polarization of majority character. For 5 nm Au, the states above $E_f$ possess a higher polarization than those below $E_f$. This difference decreases for larger Au film thicknesses. The error bars are determined as the standard deviation for negative pump-probe delays.

## Results and discussion

Figure 3 shows the spin-polarization as a function of the pump-probe delay for states above and below the Fermi energy. Depending on the thickness of the Au layer, we observe a small static polarization before the pump laser pulse. The reason for this polarization is likely the Ruderman-Kittel-Kasuya-Yosida^35^ interaction with the Fe film. Once the laser excites the ferromagnet, we observe an increase in the spin polarization to $\approx 4$ %. The pump-induced polarization has majority character for electrons below and above $E_f$. This is in contrast to the minority spin polarization of Fe at the Fermi energy^33^. The detected majority polarization is in line with the super-diffusive model^13^ as the majority electrons travel at a higher velocity. It is also in line with the thermodynamic model^36^ as the chemical potential of the majority electrons is affected more by the heating pulse compared to the minority chemical potential. For all measurements, the rise time of the spin polarization is $< 200$ fs. The measurements for 5 nm Au show a slightly higher polarization above $E_f$ than below $E_f$. This difference is less pronounced for the thicker Au layers. A possible explanation for this result is that the transport in 5 nm Au is still quasi-ballistic, whereas in the thicker films, the spin polarization within the valence band has equilibrated.

The sign of the injected spin polarization for delay times $t > 300$ fs is consistent with several theoretical predictions^20–22, 36^. However, for delay times $< 100$ fs, several authors observed the injection of minority spins into the gold layer^18, 21^. This negative polarization shortly after the excitation pulse is not present in our data. In^21^ the authors attributes this transient minority population to the difference in the lifetimes of majority and minority carriers due to the difference in kinetic energy. We expect this effect to also depend on the thickness of the Au film and the propagation within the film, which would explain the difference of our data to the reported findings.

**Figure 4:**
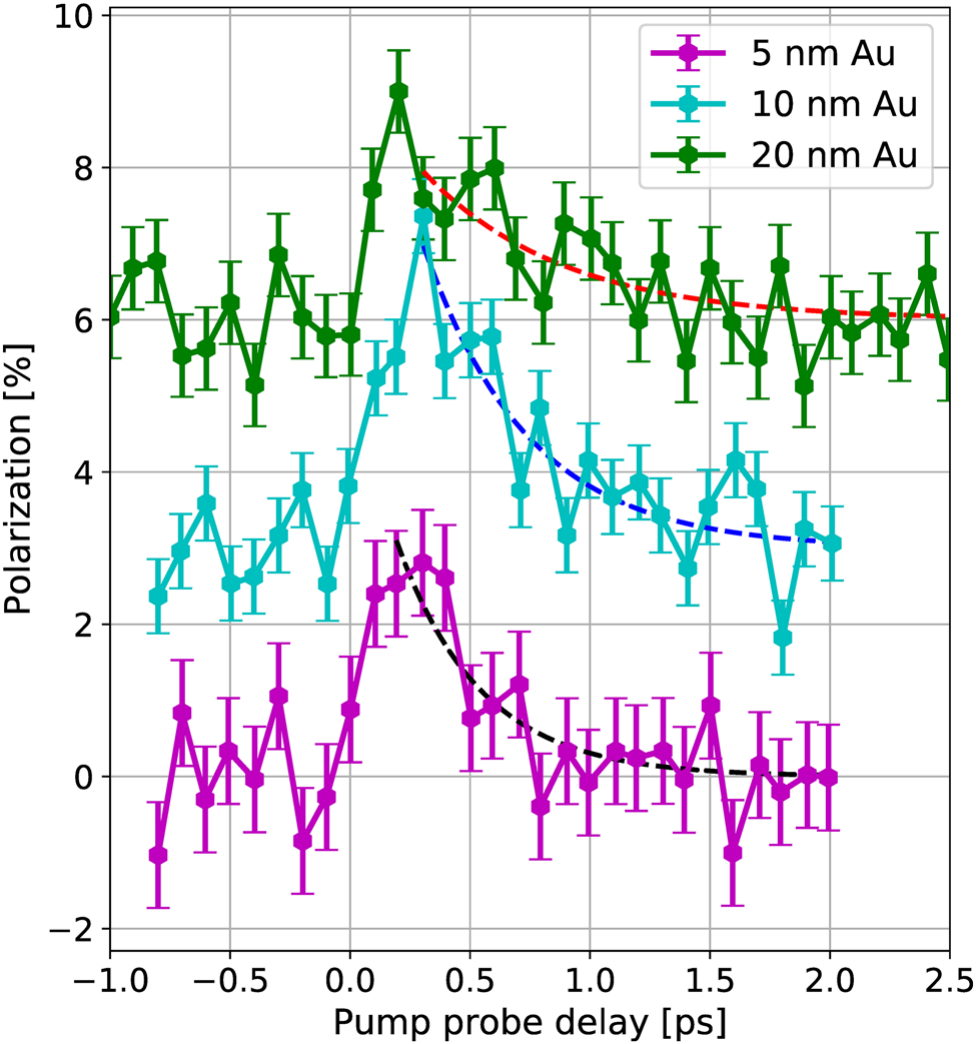
The lifetime of the spin polarization depends on the Au film thickness. The 1/e decay times are $(350 \pm 100)$ fs for 5 nm Au, $(440 \pm 70)$ fs for 10 nm Au and $(580 \pm 190)$ fs for 20 nm Au. The onset of the population of states above $E_f$ has been used to determine the exact zero-time for the exponential fit. The curves are offset by 3 % for readability. The error bars are determined as the standard deviation for negative pump-probe delays.

Figure 4 shows the spin polarization averaged over red and blue energy intervals below and above $E_f$. For all thicknesses, the spin polarization rises within $< 200$ fs (as already seen in Fig. 3). However, there is a significant difference in the decay time. The decay time $\tau (d)$ as a function of the Au film thickness *d* is determined by an exponential fit and result in 350 fs (5 nm Au), 440 fs (10 nm Au) and 580 fs (20 nm Au). The decay of the spin polarization is caused by two processes: spin flips in the bulk of the Au film as well as spin transport to the Fe/Au interface which includes subsequent flips in the Fe layer or at the interface. Thus, the decay time reads:

1 $$\begin{aligned} \frac{1}{\tau (d)} = \frac{1}{\tau _{\mathrm {sf, Au}}} + \frac{1}{\tau _{\mathrm {tr}}(d)}. \end{aligned}$$

Here, $\tau _{\mathrm {sf, Au}}$ is the spin flip scattering time in the Au film and $\tau _{\mathrm {tr}}(d)$ is the spin transport-induced relaxation time. The density of spins stored in the Au film depends on the difference in the chemical potentials for minority and majority spins $\mu _\uparrow , \mu _\downarrow$. The gold film therefore acts as a spin capacitor. The spin capacitance density^11^ can be defined as the derivative

2 $$\begin{aligned} c_s = -e\frac{\mathrm {d}(n_\uparrow - n_\downarrow )}{\mathrm {d}(\mu _\uparrow - \mu _\downarrow )}. \end{aligned}$$

Here, $n_{\uparrow ,\downarrow }$ is the carrier density for majority and minority carriers and *e* is the elemental charge. In a normal metal, the density of states is independent of the spin direction. Therefore, it only depends on the density of states at the Fermi energy $N_s$ as^11^

3 $$\begin{aligned} c_s = -e N_s. \end{aligned}$$

In this time-resolved experiment, we study a non-equilibrium situation: Strictly, the chemical potentials are not well-defined for the first 50 fs as the pump laser has generated a significant fraction of non-thermal electrons^33^. However, the non-thermal electrons (at least in the iron film) decay and are insignificant after 100 fs. In addition, the transport from the Fe layer to the Au layer causes the non-thermal electrons to further decay. Experimentally, we could not observe non-thermal electrons in the Au layer.

The spin diffusion length in Au of $\approx 50$ nm is larger than the film thickness. Therefore, we approximate that the spin voltage is constant within the Au layer. In this case, the spin capacitance per unit area is proportional to the thickness *d* of the Au layer: $C_s = e N_s d$. Changing the charge of the spin capacitance requires spin flips in the bulk (which corresponds to a leakage current within the spin capacitor) or transport to the ferromagnet. Internal spin flips lead to the constant spin decay time $\tau _{\mathrm {sf, Au}}$, whereas the thickness dependent part leads to the decay time $\tau _{\mathrm {tr}}(d)$. From the data presented in Fig. 4 we find $\tau _{\mathrm {sf, Au}} = 700\,{\mathrm {fs}}$. For the thickness dependent part $\tau _{\mathrm {tr}}(d) = \alpha d$ with $\alpha = 140$ fs/nm. The current responsible for discharging the spin capacitor depends on the cooling rate of the ferromagnetic layer as well as the conductance of the ferromagnet - gold structure. Thus, the observed thickness dependence could also be affected by changes of the cooling rate due to electron-phonon coupling within the gold layer.

Spin-resolved photoemission gives access to the spin accumulation “charge” of the spin capacitor. It is therefore complementary to the THz emission experiments, which allow for the measurement of the spin current^15, 23, 24^, that leads to charging and discharging of the spin capacitor. In such THz experiments, the spin current can be determined from the THz field emitted by the ferromagnet—normal metal sample through the spin-Hall effect.

## Conclusion

In conclusion, we perform a time-resolved spin injection experiment with spin- and time-resolved photoelectron spectroscopy. The demagnetization of a ferromagnetic iron layer causes the injection of a spin current into a gold layer where we can detect the spin polarization as a function of time.

The injected spin current is polarized along the majority spin direction, and its polarity is equal above and below the Fermi energy. This result is in contrast to the spin polarization of Fe at the Fermi edge, which is dominated by minority electrons.

The spin polarization increases on a time scale of $< 200$ fs. For a film thickness of 5 nm, we observe a higher polarization for the electrons above $E_f$. With higher film thicknesses, the polarizations below and above $E_f$ equilibrate. The pump-induced spin polarization in Au reaches approx. 4%, which is on the same order of magnitude as that observed by Hofherr et al.^19^. However, a direct comparison of both experiments is difficult as they used a Ni film on a semi-infinite Au layer whereas we used a Fe film on a thin gold layer of up to 20 nm thickness. In addition, they quenched the Ni magnetization by 87%. Furthermore, the different probing depth may affect the results as well.

The decay rate of the spin polarization within Au can be measured directly. We observe a thickness dependence, indicating that spin transport provides an important contribution to the de-polarization of Au. The capacitive aspect of spin injection dynamics may limit the speed of future spintronic devices.
